# The expression of HSP60 and HSP10 in large bowel carcinomas with lymph node metastase

**DOI:** 10.1186/1471-2407-5-139

**Published:** 2005-10-28

**Authors:** Francesco Cappello, Sabrina David, Francesca Rappa, Fabio Bucchieri, Lorenzo Marasà, Tommaso E Bartolotta, Felicia Farina, Giovanni Zummo

**Affiliations:** 1 Sezione di Anatomia Umana, Dipartimento di Medicina Sperimentale, Università degli Studi di Palermo, Italy; 2 Reparto di Anatomia Patologica, Ospedale "Civico", Palermo, Italy

## Abstract

**Background:**

The involvement of Heat Shock Proteins (HSP) in cancer development and progression is a widely debated topic. The objective of the present study was to evaluate the presence and expression of HSP60 and HSP10 in a series of large bowel carcinomas and locoregional lymph nodes with and without metastases.

**Methods:**

82 Astler and Coller's stage C2 colorectal cancers, of which 48 well-differentiated and 34 poorly-differentiated, were selected along with 661 lymph nodes, including 372 with metastases and 289 with reactive hyperplasia only, from the same tumours. Primitive tumours and both metastatic and reactive lymph nodes were studied; specifically, three different compartments of the lymph nodes, secondary follicle, paracortex and medullary sinus, were also analysed. An immunohistochemical research for HSP60 and HSP10 was performed and the semiquantitative results were analysed by statistical analysis to determine the correlation between HSPs expression and 1) tumour grading; 2) degree of inflammation; 3) number of lymph nodes involved; 4) lymph node compartment hyperplasia. Moreover, western blotting was performed on a smaller group of samples to confirm the immunohistochemical results.

**Results:**

Our data show that the expression of HSP60, in both primary tumour and lymph node metastasis, is correlated with the tumoral grade, while the HSP10 expression is not. Nevertheless, the levels of HSP10 are commonly higher than the levels of HSP60. In addition, statistical analyses do not show any correlation between the degree of inflammation and the immunopositivity for both HSP60 and HSP10. Moreover, we find a significant correlation between the presence of lymph node metastases and the positivity for both HSP60 and HSP10. In particular, metastatic lymph nodes show a higher percentage of cells positive for both HSP60 and HSP10 in the secondary follicles, and for HSP10 in the medullary sinuses, when compared with hyperplastic lymph nodes.

**Conclusion:**

HSP60 and HSP10 may have diagnostic and prognostic significance in the management of this tumour and their overexpression in tumoral cells may be functionally related to tumoral progression. We hypothesise that their expression in follicular and medullary cells of lymph nodes may be induced by formation of metastases. Further studies based on these observations could lead to a better understanding of the HSPs involvement in colorectal cancer progression, as well as other neoplasms.

## Background

Heat shock proteins (HSP) are a family of molecules that are highly conserved during evolution and involved in many cellular functions, such as protein folding. Consequently, their alteration may have multiple pathophysiologic effects and the number of papers studying their expression in normal and pathologic conditions is constantly increasing [1-3]. In particular, the role of a number of HSPs, such as HSP27, -70, -72 and -90, during carcinogenesis has already been widely investigated, *in vivo *and *in vitro*, in many conditions, such as lung [4], breast [5], esophageal [6] and ovarian [7] cancer, as well as osteosarcoma [8], and lymphoblastic leukemia [9]. The data obtained in these studies seems to suggest that this group of HSPs may be useful as tools in the management of primitive neoplasms. Some articles have also suggested a possible relationship between HSP expression and lymph node metastasis formation [10-15].

HSP60 and HSP10 are two chaperones that interact in a two-step folding mechanism in the mitochondria of prokaryotic and eukaryotic cells [16]. In addition, these proteins may be involved in other cellular functions, such as mediating specific tumour signals, but these roles are not yet well understood [3]. In the last few years, our research group has evaluated the presence and expression of HSP60 and HSP10 in a series of carcinogenetic models, such as the "dysplasia-carcinoma" sequences of uterine exocervix [17,18], large bowel [18,19] and prostate [20]. These data have highlighted that these chaperones are overexpressed during the carcinogenetic steps; in particular, they accumulate in the cytoplasm of dysplastic and neoplastic cells, and their levels of expression increases in the sequence leading from dysplasia towards carcinoma. We have hypothesised that HSP60 and HSP10 might be considered as new diagnostic and prognostic tools for these cancers [21,22], being involved in the molecular steps of carcinogenesis, analogously to what has already been demonstrated with other tumours [23-28].

HSP10 was recently shown to be selectively expressed by myelocyte and megakaryocyte precursors in normal human bone marrow [29]. This feature disappears during lineage maturation, and it was hypothesised that HSP10 might have another role during differentiation and/or proliferation of those normal cellular lineages apart from the co-chaperonin one, although the obtained results could not explain this selective expression.

In view of these factors, in the present work the presence and expression of HSP60 and HSP10 were studied in a series of advanced *large bowel carcinomas *(LBC) with lymph node metastases. In particular, we investigated whether their expression was dependent on the grade of differentiation of the tumour and presence of regional lymph node metastasis. Moreover, we analysed the significance of the data to determine the correlation between HSP expression and both degree of inflammation and number of lymph nodes involved. Finally, three different compartments of each reactive lymph node, the secondary follicles (SF), the paracortex (PC) and the medullary sinuses (MS), were examined; lymph nodes with metastases (MLN) were compared with lymph nodes with reactive hyperplasia (HLN) only, to determine any differences in HSP60 and HSP10 expression.

## Methods

### Immunohistochemistry

82 LBC of Astler and Coller's stage C2, with locoregional node metastases, were collected. 48 *well-differentiated *(G1) carcinomas were compared with 34 *poorly-differentiated *(G3) ones. The specimens were formalin-fixed and paraffin-embedded. From each case, a 5 micra section of both *tumour infiltrating the bowel wall *(Ti) and *lymph node with metastasis *(Ni) were obtained. 10 specimens of normal colonic mucosae were selected, from which 5 micra sections were obtained as controls.

From all the tumours collected, 661 lymph nodes were selected and divided in two groups; the first comprising of 372 lymph nodes with metastases (MLN) and the second of 289 lymph nodes with reactive hyperplasia (HLN) only. A 5 micra section was obtained from each sample.

An immunostaining for HSP60 (monoclonal, SIGMA, H4149, 1:400), and HSP10 (Polyclonal, StressGen, SPA-110, 1:400) was performed on the Ti, Ni, MLN and HLN sections, using an avidin-biotin complex kit (LSAB2, DAKO, Cat. No. K677). A non-immune serum was run concurrently as negative control. 3-3'-diaminobenzidine (DAB chromogen solution, DAKO, Cat. No. K3467) was used as develop chromogen. Nuclear counterstaining was obtained using hematoxylin (DAKO, Cat. No. S2020).

Three independent observers (F.C., F.R. and L.M.) examined the specimens and performed a semiquantitative analysis to evaluate the percentage of positive cells in 10 HPF. The mean of the triplicate observation data was used for statistical analyses. Moreover, we semiquantified the degree of inflammation (infiltrating lymphocytes) in each tumoral specimen on a scale of 0–3 + (from absent to strongly positive).

We analysed the significance of the data using the Student "t" test (P < 0.05). A one-way "analysis of variance" (ANOVA) was used to determine the correlation between HSP expression and 1) tumour grading; 2) degree of inflammation; 3) number of lymph nodes involved; 4) lymph node compartment hyperplasia.

### Western blotting

Biopsies from specimens of Ti were frozen in liquid nitrogen for molecular biology. Specifically, 10 G1 and 10 G3 specimens of both tumours and metastatic lymph nodes were collected randomly, along with 4 biopsies of normal colonic mucosae as controls.

20 μg of total tissue extracts in each lane and a protein marker (Kaleidoscope prestained standard, Bio-Rad, Cat. No 1610324) were separated by electrophoresis on denaturing 15% polyacrylamide slab gel (SDS-PAGE) and transferred to a nitrocellulose membrane (Nitrocell Paper, Bio-Rad, Cat. No 1620115). After 1 hour at room temperature (RT) with a blocking buffer (5% low-fat dried milk in TBST: 50 mM Tris-HCl pH 7,5, 150 mM NaCl, 0,1 % Tween-20) under gentle shaking, the membrane was incubated with anti-HSP60 primary antibody (monoclonal mouse, SIGMA, H4149, 1:1000) overnight at 4°C. After washings, the membrane was incubated with HRP-conjugated secondary antibody (anti-mouse, Pierce, 1:10000, Cat. No 31432) for 1 hour at RT with shaking and the specific binding was detected using a chemiluminescent substrate (SuperSignal West Pico Chemiluminescent Substrate, Pierce, Cat. No 34080) for autoradiography.

The same membrane was stripped with a stripping buffer (Restore TM Western Blot Stripping Buffer, Pierce, Cat. No 21059) and incubated with anti-HSP10 primary antibody (polyclonal rabbit, StressGen, SPA-110, 1:2000), following the procedures described above (secondary antibody: anti-rabbit, Pierce, 1:20000 Cat. No 31462).

## Results

### HSP60 and HSP10 positivity in primitive versus metastatic tumours of different grade

**Figure 1:**
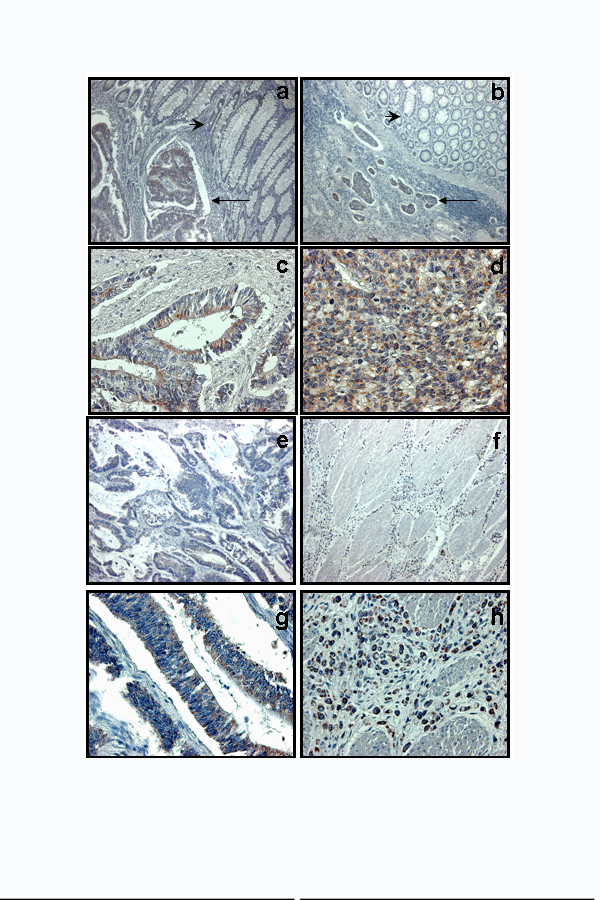
HSP60 positivity in carcinoma (arrow) but not in normal tissues (arrowhead) in specimens from both well differentiated (a) and poorly differentiated (b) tumours (Magnification: 10×). A higher magnification (40×) shows that the positivity is diffuse into cytoplasm of tumoral cells of both G1 (c) and G3 (d) specimens; few positive interstitial (inflammatory) cells were scattered in the interposed stroma. HSP10 is also diffusely expressed by tumoral cells of both G1 (e) and G3 (f) LBC (Magnification: 10×). A higher magnification (40×) of both G1 (g) and G3 (h) shows that the positivity is mainly localised in the cytoplasm of neoplastic elements.

HSP60 was present in 40 out of 48 (83.3%) Ti G1 (fig. 1a,c) and in 32 out of 34 (94.1%) Ti G3 LBC (fig. 1b,d), while HSP10 was present in all examined specimens of Ti of both G1 (fig. 1e,g) and G3 (fig. 1f,h) LBC. In these specimens, both molecules were present in the cytoplasm of neoplastic cells, and they were also rarely present in some inflammatory elements scattered in the stroma. In particular, statistical analyses, we did not find any correlation between the degree of inflammation and the immunopositivity for HSP60 (p > 0.05) and HSP10 (p > 0.1).

**Figure 2:**
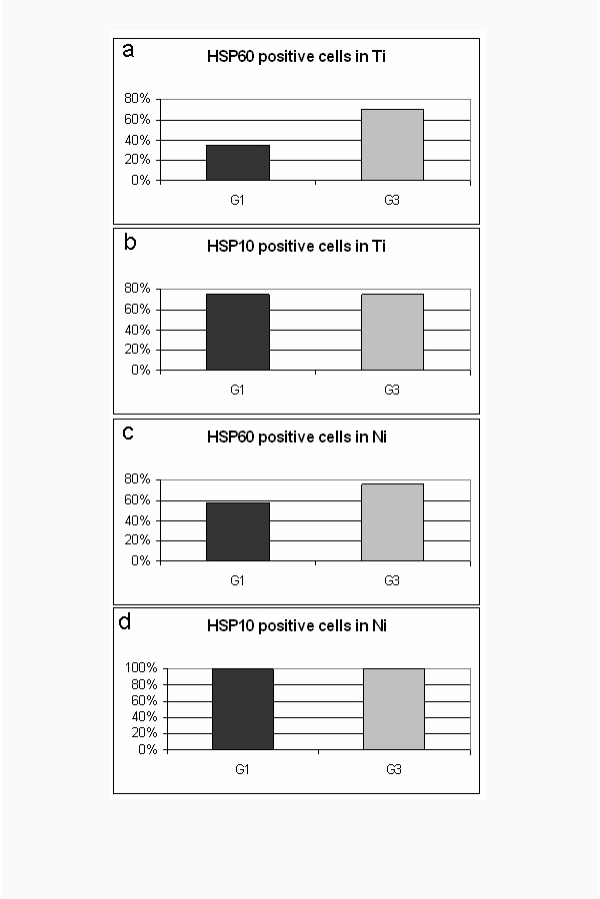
Graphics showing comparison between data obtained by quantitative analyses of HSP60 (a) and HSP10 (b) positive tumoral cells in G1 and G3 Ti and Ni.

In addition, the percentage of HSP60 positive cells was higher in the G3 group (mean: 70%) compared with the G1 (mean: 35%) (fig. 2a), while a similar number of HSP10 positive elements were present in both groups (mean: respectively 74% and 75%) (fig. 2b). Normal epithelium above the infiltrating neoplasms resulted commonly negative or with few scattered positive elements (fig. 1b), similarly to what observed in the biopsies of normal colonic mucosae (data not shown). Statistical analyses showed that the difference between the number of HSP60 positive cells in G1 and G3 LBC was significant (p < 0.0005), while statistic difference was not found in HSP10 positivity (p > 0.05).

**Figure 3:**
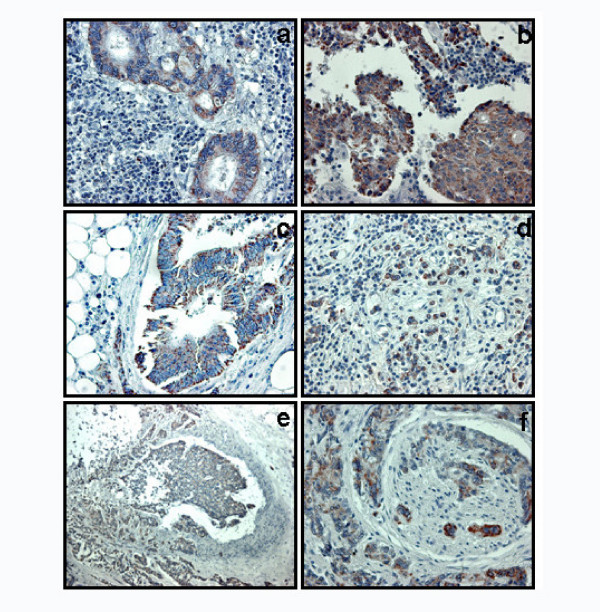
Infiltrated lymph nodes from G1 (a) and G3 (b) LBC show tumoral glands positive for HSP60. Metastases also show glands positive for HSP10 in both G1 (c) and G3 (d) carcinomas (Magnification: 40×). HSP60 positivity shows vascular (e) and neural (f) invasion by cancer (Magnification: 10×).

Fig. 2c shows that 28 out of 48 Ni of G1 LBC (58%) were positive for HSP60 (fig. 3a), compared to 26 out of 34 Ni of G3 CRC (76%) (fig. 3b). Fig. 2d shows that all Ni of both G1 (fig. 3c) and G3 (fig. 3d) LBC were positive to HSP10. Statistical analyses showed that the difference between the number of HSP60 positive Ni in G1 and G3 LBC was significant (p < 0.01), while statistic difference was not found for HSP10 positivity (p > 0.05). Both HSP60 and HSP10 positivity was often co-localised within vascular (fig. 3e) and nervous (fig. 3f) structures invaded by neoplastic tissue in both Ti and Ni.

**Figure 4:**
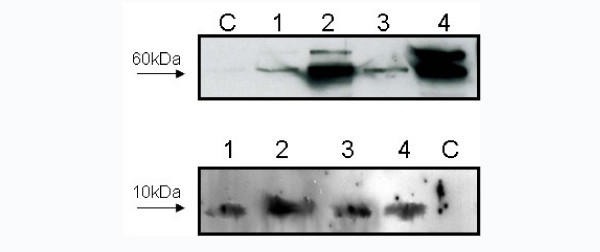
Western blot analyses for the research of HSP60 and HSP10 in tissue extracts of normal colonic mucosa (1), Ti G1 (2), Ti G3 (3), Ni G1 (4) and Ni G3 (5).

The results of the immunoblotting analyses were comparable to the immunohistochemical data (fig. 4). The quantity of HSP60 was higher in G3 specimens of both Ti and Ni, when compared to G1. HSP10 was present in a similar amount in all examined specimens. Specimens of normal colonic tissue were commonly under the threshold of detectability for both HSP60 and HSP10.

### HSP60 and HSP10 positivity in metastatic versus hyperplastic lymo nodes

We selected 661 lymph nodes from all the tumours (mean: 8.1; range: 5–12; S.D. 2.2) and divided in two groups; the first comprising of 372 MLN and the second 289 HLN. Firstly, we examined the presence of a statistic correlation between HSPs expression and number lymph node involved by the disease. We found the presence of a significant correlation between the presence of metastases and the positivity for both HSP60 (p < 0.005) and HSP10 (p < 0.001) in lymph nodes.

**Figure 5:**
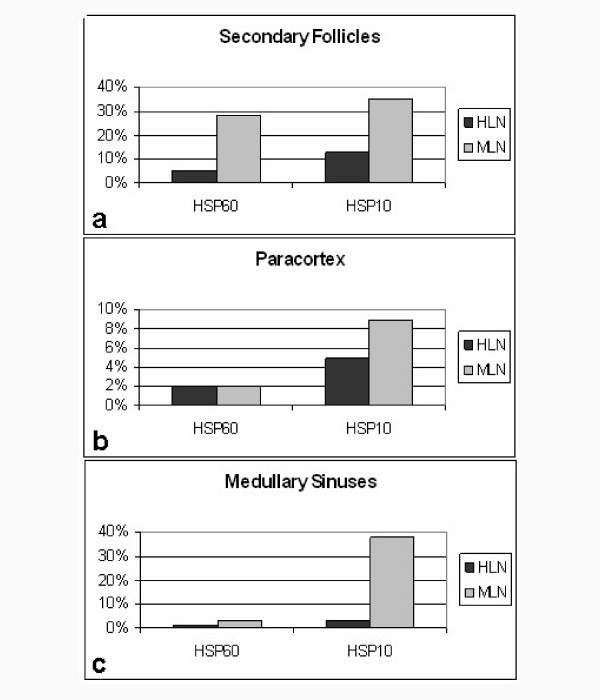
Diagrams showing the differences of the mean number of positive cells between HLN and MLN in the lymph node compartments: a) secondary follicles; b) paracortex; c) medullary sinuses.

Subsequently, a semiquantitative analysis on the immunohistochemical observations in the lymph node compartments of HLN was performed. Table 1 summarizes these results. In SF cells of the HLN group, only 5% were positive for HSP60 and 13% for HSP10. On the contrary, we found an increase in the number of HSP60 (28%) and HSP10 (35%) positive cells in SF of MLN (fig. 5a). We also found a great increase in the number of HSP10 positive cells in MS cells (38%) of MLN when compared to the HLN group (3%). There was no significant difference in the number of HSP60 positive cells between both HLN and MLN groups in MS (fig. 5b), similarly to the number of cells positive to both chaperones in PC of both HLN and MLN groups (fig. 5c). Statistical analyses showed a significant difference between the number of HSP60 positive cells in SF of the HLN and MLN groups (p < 0.0003).

**Table 1:** Mean percentages and ranges of immunopositive cells

**HLN**	**PART**	**MEAN**	**RANGE**	**MLN**	**PART**	**MEAN**	**RANGE**
	*SF*	5%	0–8%		*SF*	28%	7–41%
**HSP60**	*PC*	2%	0–4%	**HSP60**	*PC*	2%	0–4%
	*MS*	1%	0–2%		*MS*	3%	0–6%
							
	*SF*	13%	4–22%		*SF*	35%	18–58%
**HSP10**	*PC*	5%	2–8%	**HSP10**	*PC*	9%	4–19%
	*MS*	3%	1–5%		*MS*	38%	22–65%

Mean percentages and ranges of immunopositive cells in hyperplastic lymph nodes (HLN), left side, and metastatic lymph node (MLN), right side. SF: secondary follicles; PC: paracortex; MS: medullary sinuses. See text for more details.

**Figure 6:**
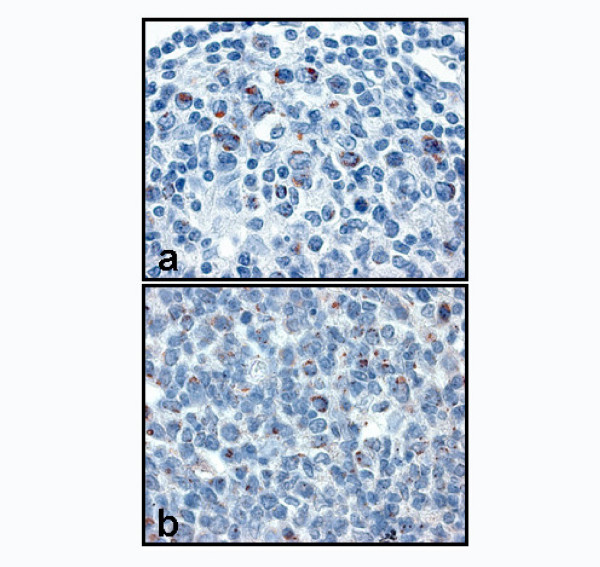
High magnifications (100×) of immunostaining for HSP60 (a) and HSP10 (b) show cytoplasmic positivity.

Analogously, the number of HSP10 positive cells in both SF (p < 0.001) and MS (p < 0.0001) presented a significant difference. The positivity for both HSP60 (fig. 6a) and HSP10 (fig. 6b) in the cells of all reactive lymph node compartments was commonly localised in the cytoplasm.

## Discussion

Although HSPs were first defined as proteins induced by environmental and pathophysiologic stress, they are also implicated in protein-protein interactions, such as folding, translocation, and prevention of inappropriate protein aggregation. Recently, other functions concerning their pivotal roles during cancer development and progression have been suggested (30).

In a study conducted on a series of esophageal squamous cell carcinomas, the overexpression of HSP70 was correlated with lymph node metastasis, and lymphatic vessel invasion, and the authors suggested that HSP70 expression might be used to assess the clinical outcome after surgery [12]. A reduced expression of HSP70 and HSP40 has also been associated with a lower histopathologic differentiation in a series of gastric carcinomas [31]. In addition, Hwang et al. [13] have demonstrated that the expression of HSP70 and HSP110 was increased in highly metastatic colorectal cancer cell lines, but not in weak metastatic cells, suggesting that the expression of these HSPs is highly correlated with the advanced clinical stages and positive lymph node involvement. In multivariate analyses concerning the type, grade, stage of the tumour, invasion of lymphatics, blood vessels and nerves as well as lymph nodes, in 36 pancreatic adenocarcinomas, HSP70 immunoexpression was found to be an independent prognostic factor [32]. Finally, in an immunohistochemical study on 102 esophageal squamous cell carcinoma specimens Kawanishi et al. [33] suggested that the expression of HSP27 and HSP70 was frequently reduced and therefore it should be considered an independent prognostic factor in this disease.

In their study on primary invasive ductal carcinomas of the breast with lymph node metastasis, Storm et al. [11] found that HSP27 might confer cytoprotection for metastatic cells, and they postulated that HSP27 overexpression is associated with reduced disease-free survival in breast carcinomas. Contrastingly, Tetu et al. [34] did not find any predictive role for HSP27 in the outcome in node-positive breast carcinomas.

Piselli et al. [35] recently performed a cytofluorimetric analysis on human pancreatic adenocarcinoma cells, both grown in vitro and collected ex vivo from primary tumours or lung metastases of tumour-engrafted SCID mice; they were the first to demonstrate an HSP60 surface expression on metastatic cells, but this expression was not correlated with metastasization. In a multivariate analysis on a series of metastatic breast cancers, Schneider et al. [36] demonstrated that neither HSP27 nor HSP60 expression was able to exclude axillary node invasion completely. Finally, Ito et al. [37] studied the expression of HSP27, -60, -70 and -90 in 24 squamous cell carcinomas of the tongue using immunohistochemistry, finding that HSP immunoexpression might change during tumorigenesis, but there was no correlation between HSP staining and survival period, clinical stage, lymph node metastasis, histological grade or p53 immunostaining.

As far as we are aware, the present work is the first study reporting the expression of HSP60 and HSP10 in a series of LBC with lymph node metastases, and the immunolocalisation of these molecules in the different compartments of reactive lymph nodes.

The presence and expression of HSP60 and HSP10 in some carcinogenic models, in particular, both pre-tumoral (dysplastic) and neoplastic lesions of large bowel, as well as uterine exocervix and prostate gland has been investigated previously [17-20]. These experiments showed that the level of these two strictly related mitochondrial chaperonins increases from normal through dysplastic towards neoplastic tissues. These proteins resulted diffusely localised in the cytoplasms of dysplastic and neoplastic cells. Moreover, few scattered inflammatory elements were occasionally positive at stromal level. Considering this overexpression, it was hypothesised that these molecules might have different functions during cancer development, apart from the mitochondrial regeneration during normal cell proliferation. Nevertheless, the exact nature of this role is still not understood.

In the present paper, the expression of HSP60 in Ti and Ni was found to be dependent on the tumoral grade, while the expression of HSP10 was not. The number of HSP60 positive tumoral cells in Ti of G3 LBC was higher than in G1, and the number of HSP60 positive Ni in G3 LBC was higher than in G1. Immunohistochemical results were confirmed by immunoblotting analysis. Therefore, we postulate a prognostic significance of these data. HSP10 was strongly positive in all examined specimens, and these results may have a diagnostic utility. Interestingly, a higher positivity for HSP60, but not for HSP10, was correlated with the presence of lymph node metastasis and this data may have a histopathologic value.

Although normal epithelia periodically regenerate cellular elements by mitosis of basal cells, the present work shows that HSP60 and HSP10, in normal epithelia, are under the antibody detection threshold for immunohistochemical analyses, while neoplastic elements show a strong cytoplasmic expression of these proteins. Many papers have already shown the involvement of the HSP60/HSP10 complex in preventing the activation of the apoptotic machinery. Samali et al [38] were the first to demonstrate that pro-caspase-3 is present in the mitochondrial fraction of Jurkat T cells in a complex with the chaperon proteins HSP60 and HSP10 and that the release of mitochondrial HSP may also accelerate caspase activation in the cytoplasm of intact cells. These data are in accordance with the findings of Xanthoudakis et al. [39] who showed that ATP-dependent 'foldase' activity of HSP60 may induce pro-caspase-3 maturation in Jurkat cells stimulated to undergo apoptosis by a Fas-independent pathway and that this represents an important regulatory event in apoptotic cell death. Lin et al. [40] suggested that overexpression of the combination of HSP60 and HSP10 and of HSP60 or HSP10 individually may protect myocytes against apoptosis in an in vitro model of ischemia/reperfusion injury. More recently they have demonstrated that HSP10 overexpression may inactivate Raf, ERK, and p90Ribosomal kinase (p90RSK), suggesting that only HSP10 is involved in the complex mechanisms that protect myocytes against simulated ischemia and reoxygenation induced death.

We could postulate that both HSP60 and HSP10 are up-regulated in cancer for extramitochondrial functions, i.e. in the block of the apoptotic machinery that usually takes place during cancer development and progression. Although HSP60 and HSP10 should be functionally correlated, HSP10 is present in a higher number of specimens and with a higher expression than HSP60. This result may indicate a different function of HSP10 inside the cytoplasm of tumoral cells. In a recent study, where the expression of HSP10 and HSP60 was investigated in a series of normal human bone marrows, similar data were found [29]. Interestingly, a selective preference of HSP10 for myeloid and megakaryocytic precursors was discovered [29]. Therefore, other roles of HSP10 apart from the co-chaperonin one during bone marrow cell proliferation and differentiation of normal cells could be hypothesised. This is backed up by other studies on this co-chaperonin [41,42].

In the present study, the presence and localisation of HSP60 and HSP10 in a series of human lymph nodes were evaluated. Lymph node functioning is crucial for an individual's survival. Normal lymph nodes are very small structures, not always clinically detectable in human body. They react to antigens by uptaking and processing them, eventually destroying them. Reacting lymph nodes enlarge due to immunologic stimulation that drives their hyperplasia. Examining a histological section of hyperplastic lymph node, different compartments can be distinguished: 1) the follicles, where precursors of *plasma cells *and *memory B cells *are formed; 2) paracortex, where specific cellular response takes place, generating *antigen-specific T cells*; 3) medullar sinuses, where the lymph carried from afferent to efferent lymphatic vessels is cleared by *macrophages *[43-45]. Commonly, hyperplasia involves all lymph node structures; as already demonstrated, in a study on a wide series of HLN and MLN from axilla, the most common pattern of hyperplasia is the mixed type (66.5% of 996 HLN and 68.6% of 4711 MLN). In particular, hyperplasia mainly involved both follicles and paracortex area (22.2% of MLN and 19.2% of HLN), although often sinuses were also found enlarged [46]. A fundamental step during the examination of an enlarged lymph node is the distinction between a reactive hyperplasia and a neoplasm. The latter may be of primary (lymphoma) or secondary (metastasis) type. In an investigation on a series of 1159 lymph nodes from breast cancer, micrometastases (involving less that 25% of the lymph nodal volume) have been shown often to be present also in very small nodes (less that 2 mm in diameter) [47]. Unfortunately, the detection of metastases in a lymph node is difficult if they are small in dimension. Several studies have shown that a number of histologically negative lymph nodes may present, at a retrospective analysis, a micrometastasis revealed only immunohistochemically or with immunofluorescence techniques; the presence of micrometastasis in the lymph node has great prognostic and therapeutic implications [48-53].

In this study, the number of SF cells positive for both HSP60 and HSP10 increased significantly in MLN, when compared to HLN. We postulate that this increase may be related to formation of metastases. As a consequence, although cytokeratin staining is a much easier way to detect micrometastasis, the statistically significant increment in the MLN group of the number of HSP60 and HSP10 positive elements in secondary follicles could also be considered diagnostic to predict the presence of lymph node metastases. Therefore, a lymph node with germinal centre presenting a strong staining for HSP60 and/or HSP10 without any apparent metastasis should be examined in detail, since this observation may reflect an increased likelihood of finding a micrometastasis.

Since MS of MLN showed a significantly higher amount of HSP10, but not HSP60, positive cells, when compared to the MS of HLN, we could assume that HSP10 in this site is under unknown stimulation inducing its overexpression, for functional roles, i.e. cell proliferation. The role of HSP60 during proliferation and differentiation of eukaryotic cells has already been demonstrated [54-56]. Moreover, HSP60 and HSP10, working together, could protect mitochondrial function and prevent apoptotic cell death [37,39] although some studies have shown that these molecules do not always act as a single functional unit in vivo [41,57].

## Conclusion

Many papers have been focusing on the role of some HSPs to predict cancer progression [11-13,58]. We found particularly interesting the paper of Storm [11], who first showed the expression of HSP27 in metastatic lymph nodes to confer cytoprotection for metastatic cells of breast carcinoma and its association with the reduced disease-free survival. In addition, a selective expression of HSP70 in the germinal centres of HLN has been demonstrated, although the meaning of this overexpression is not understood [59].

Our researches showed an overexpression of HSP60 and HSP10 in LBC with lymph node metastases. Both molecules could be useful for histopathologic diagnosis of this neoplasm, as well as to better assess the prognosis. We could also assume that both proteins are involved in LBC progression, i.e. exercising an antiapoptotic effect. We hypothesise that the increased expression of HSP60 and HSP10 in reactive lymph node cells is due to their role in proliferation of normal cells. Recently it has been shown that HSP60 activates macrophages and T-cell, and this could also be a possible explanation for its overexpression in lymph nodes with metastatic cancer upregulation [60]. Based on these considerations, it could be postulated that paracrine factors, such as cytokines, may induce an up-regulation of these molecules, but the exact role of the overexpression remains to be confirmed.

Serial sectioning could be used to further study if any of the HLN population that present HSP60 and HSP10 staining in the range of MLN contain occult metastases; it could also be interesting to compare HLN from LBC specimens with non-neoplastic setting ones, i.e. resections of active inflammatory bowel disease, to confirm whether the former could be used as proper controls. Finally, the selective overexpression of HSP10 in MS of MLN could support the hypothesis that this molecule, following unknown biological stimulations, acts independently from HSP60.

In conclusion, our study could add new data to the classic classification of G1 or G3, since both HSP60 and HSP10 positivity may help to detect more aggressive tumors. Moreover, the comparison of the expression levels of these chaperones with other predictors of survival, as grading, number of metastatic lymph nodes and degree of tumor infiltrating lymphocytes may be useful in colon cancer management.

## Abbreviations

HSP: Heat Shock Proteins

LBC: Large Bowel Carcinomas

SF: Secondary Follicles

PC: Paracortex

MS: Medullary Sinuses

MLN: Lymph Nodes with Metastases

HLN: Reactive Hyperplasia

G1: Well-Differentiated Tumours

G3: Poorly-Differentiated Tumours

Ti: Specimens of Tumour Infiltrating the Bowel Wall

Ni: Specimens of Lymph Node with Metastasis

## Competing interests

The author(s) declare that they have no competing interests.

## Authors' contributions

FC designed the study, examined the immunohistochemical results, performed a semiquantitative analysis and drafted the manuscript;

SD collected of specimens, carried out the immunohistochemistry and carried out the Western blotting analyses;

FR collected of specimens, examined the immunohistochemical results and performed a semiquantitative analysis;

FB participated in the design of the study and in the draft of the manuscript, performed the statistical analysis and examined the Western blotting results ;

LM participated in the collection of specimens, examined the immunohistochemcal results and performed a semiquantitative analysis;

FF participated in the and examination of the Western blotting results and in the draft of the manuscript;

GZ coordinated the design and execution of the study.

All authors red and approved the final manuscript.

## Pre-publication history

The pre-publication history for this paper can be accessed here:


